# Organic Semiconductors Processed from Synthesis‐to‐Device in Water

**DOI:** 10.1002/advs.202002010

**Published:** 2020-09-21

**Authors:** Aiman Rahmanudin, Raymundo Marcial‐Hernandez, Adibah Zamhuri, Alex S. Walton, Daniel J. Tate, Raja U. Khan, Suphaluk Aphichatpanichakul, Andrew B. Foster, Sebastian Broll, Michael L. Turner

**Affiliations:** ^1^ Organic Materials Innovation Centre Department of Chemistry University of Manchester Oxford Road Manchester M13 9PL UK; ^2^ Photon Science Institute and the Department of Chemistry Alan Turing Building University of Manchester Oxford Road Manchester M13 9PY UK

**Keywords:** conjugated polymers, mini‐emulsion polymerization, nanoparticles, organic field‐effect transistors, Suzuki‐Miyaura coupling

## Abstract

Organic semiconductors (OSCs) promise to deliver next‐generation electronic and energy devices that are flexible, scalable and printable. Unfortunately, realizing this opportunity is hampered by increasing concerns about the use of volatile organic compounds (VOCs), particularly toxic halogenated solvents that are detrimental to the environment and human health. Here, a cradle‐to‐grave process is reported to achieve high performance p‐ and n‐type OSC devices based on indacenodithiophene and diketopyrrolopyrrole semiconducting polymers that utilizes aqueous‐processes, fewer steps, lower reaction temperatures, a significant reduction in VOCs (>99%) and avoids all halogenated solvents. The process involves an aqueous mini‐emulsion polymerization that generates a surfactant‐stabilized aqueous dispersion of OSC nanoparticles at sufficient concentration to permit direct aqueous processing into thin films for use in organic field‐effect transistors. Promisingly, the performance of these devices is comparable to those prepared using conventional synthesis and processing procedures optimized for large amounts of VOCs and halogenated solvents. Ultimately, the holistic approach reported addresses the environmental issues and enables a viable guideline for the delivery of future OSC devices using only aqueous media for synthesis, purification and thin‐film processing.

## Graphical abstract

An environmentally benign cradle‐to‐grave process from synthesis‐to‐device is demonstrated for high performance organic field‐effect transistors. This holistic approach uses aqueous processes from mini‐emulsion polymerization to purification and thin‐film deposition. Compared to conventional approaches, the process requires fewer steps, lower reaction temperatures, a significant reduction in the use of volatile organic compounds and avoids toxic halogenated solvents.

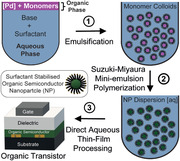

Organic semiconductors (OSCs) are critical materials for a wide range of next‐generation electronic and energy devices including thin‐film transistors,^[^ ^1^ ^]^ solar energy convertors,^[^ ^2^ ^]^ thermoelectric devices,^[^ ^3^ ^]^ chemical sensors,^[^ ^4^ ^]^ biomimetics^[^ ^5^ ^]^ and bioelectronics systems.^[^ ^6^ ^]^ The molecular properties of the OSCs (e.g., ionic and electronic charge transport, light absorption and emission, and mechanical flexibility) can be tuned by chemical design and synthesis,^[^ ^7^ ^]^ and they can be processed from solution into thin films by scalable methods suitable for mass manufacture.^[^ ^8^ ^]^ The conventional route to obtain high performance OSC devices involves the use of large quantities of volatile organic compounds (VOCs), in particular toxic halogenated solvents (e.g., chloroform, and chlorobenzene) during the synthesis, purification and device processing of these materials.^[^ ^9^ ^]^ Halogenated solvents are generally considered essential to purify and solution‐process these materials, as they can impart high solubility and the self‐assembly properties in thin films necessary for achieving high performance in a device. However, concerns about the environmental and health impact of these methods are hampering the potential commercial opportunities.^[^ ^10^ ^]^ Considering the importance of OSCs in future electronic and energy technologies, the key to delivering the promise of truly scalable and printable materials is the urgent need to develop safer, sustainable and more environmentally benign synthesis‐to‐device processes that reduce or avoid the use of toxic VOCs without sacrificing the ultimate device performance.

The conventional approach to realize an OSC device consists of multiple processes as depicted in **Figure** **1**a. The synthesis of most semiconducting polymers relies on metal catalysed cross‐couplings such as Suzuki‐Miyaura, Stille, or direct arylation step‐growth polycondensation reactions.^[^ ^11^ ^]^ These reactions generally require high temperatures (≥90 °C), and energy intensive purification steps that use large amounts of VOCs and halogenated solvents, such as preparative size‐exclusion chromatography or soxhlet extraction to deliver the purified polymer for solution processing into devices.^[^ ^12^ ^]^ In device fabrication, the polymer is dissolved in a good solvent (typically a halogenated solvent) that is used to deposit the semiconducting thin film. Previous studies have shown that OSC thin films can be deposited using less‐toxic non‐halogenated organic solvents (e.g., xylene, tetralin, THF and alcohols),^[^ ^9^, ^13^ ^]^ and solvent‐free melt deposition techniques.^[^ ^14^ ^]^ However, these studies required extensive screening and optimization of processing variables to replicate the device performance typically obtained for semiconducting thin films processed using halogenated solvents. Synthetic approaches have been used to tune the molecular structure of the OSC to improve solubility and processability with less toxic organic solvents,^[^ ^15^ ^]^ and even in water.^[^ ^16^ ^]^ Though, these chemical modifications generally require additional synthetic steps that increase solvent usage and energy consumption in the overall synthesis‐to‐device process.

**Figure 1:**
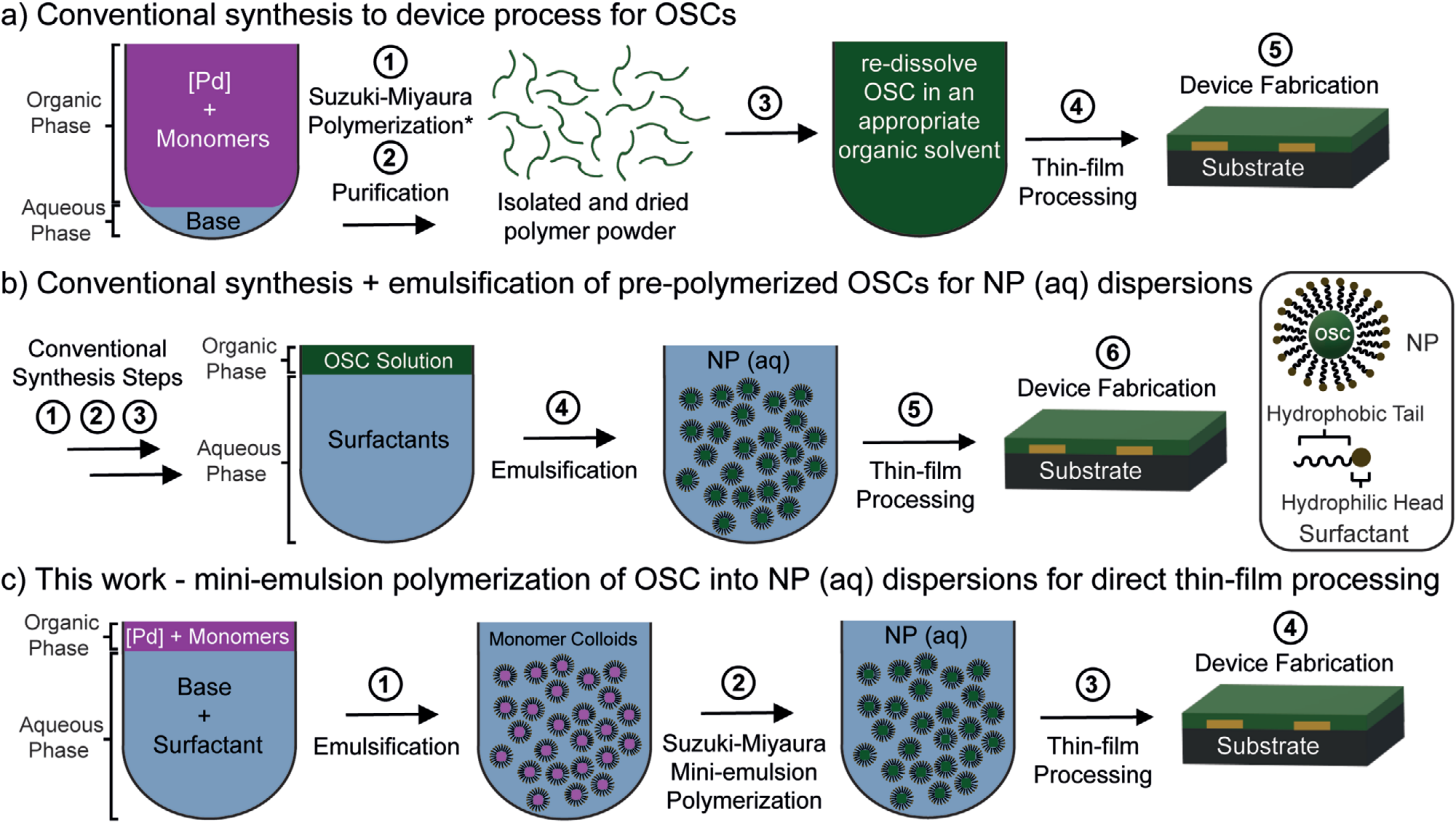
Simplified schematic description of the overall synthesis‐to‐device process for OSCs: a) Conventional process: 1) Representative metal‐catalyzed cross‐coupling polymerization of semiconducting polymers via biphasic Suzuki‐Miyaura reaction conditions (:alternatively Stille or direct arylation reactions); 2) Purification using organic solvent processes (Soxhlet or preparatory size‐exclusion chromatography); 3) Dissolving of semiconducting polymers in halogenated solvents; 4) Solution‐processing into thin films and deposition of relevant electrodes. Preparation process for NP dispersions of OSCs in water: b) Conventional synthesis and purification (steps 1–3 from 1a) + emulsification of the pre‐polymerized OSC followed by thin film deposition from water; c) Mini‐emulsion polymerization via Suzuki‐Miyaura coupling for direct thin‐film processing (this work).

Commercially important conducting polymers, such as poly(3,4‐ethylenedioxythiophene) (PEDOT) stabilized by a poly(styrenesulfonate) (PSS) counter‐ion, have been routinely processed into thin films from aqueous dispersions as they are generally synthesized in water by an oxidative polymerization reaction.^[^ ^17^ ^]^ Aqueous PEDOT:PSS dispersions can be directly processed into robust conducting thin films that have been extensively utilized as electrodes or electrode modifying layers in a variety of electronic devices.^[^ ^18^ ^]^ Extension of this concept to the processing of intrinsically semiconducting polymers is not yet well‐established, but offers an attractive prospect as coupling of this approach with the established aqueous‐processing of conductive materials on sustainable, flexible substrates could lead to a significantly more environmentally benign process.^[^ ^19^ ^]^

To date, semiconducting polymers have been dispersed as nanoparticles (NPs) in an aqueous medium for use in biomedical applications such as bio‐imaging, cell activity regulation, and photodynamic therapy.^[^ ^20^ ^]^ Recent reports have adapted this approach to prepare aqueous NP dispersions that are based on high performance semiconducting polymers, as a promising environmentally benign solution‐processing method for organic field‐effect transistors (OFETs),^[^ ^21^ ^]^ organic photovoltaics,^[^ ^22^ ^]^ and organic light‐emitting diodes.^[^ ^23^ ^]^ These NP dispersions are typically prepared by emulsifying a pre‐polymerized material, dissolved in a good organic solvent (typically halogenated solvents), into a continuous aqueous phase using a surfactant, followed by evaporation of the organic solvent to generate a colloidally stable NP dispersion (see Figure 1b). The advantage of using aqueous‐processed semiconducting polymer thin films is clearly identified in these reports, but the current methodology still requires halogenated solvents to ensure good solubility of the polymer for uniform NP dispersion and conventional polymer synthesis and purification in an organic solvent before emulsification into a colloidal dispersion.

Alternatively, semiconducting polymers can be prepared directly as aqueous dispersions using a mini‐emulsion reaction, where the dispersed organic phase contains the starting conjugated monomers and reagents (e.g., catalyst, ligands) and these are polymerized in a continuous aqueous phase to give a NP dispersion of the semiconducting polymer directly (see Figure 1c).^[^ ^24^ ^]^ Generally, the Suzuki‐Miyaura coupling reaction is used as this reaction is known to be robust when conducted in aqueous media.^[^ ^25^ ^]^ The NP dispersions prepared by this approach have largely been used to investigate NP self‐assembly and optical behavior,^[^ ^26^ ^]^ or as stable colloidal dispersions for use in photocatalytic applications.^[^ ^27^ ^]^ However, the preparation of high‐performance semiconducting polymers by a mini‐emulsion polymerization to form aqueous NP dispersions for direct processing into semiconducting thin films for OSC‐based electronic devices has yet to be demonstrated.

Herein, we demonstrate high performance p‐and n‐type OFETs fabricated by direct processing of thin films from aqueous NP dispersions prepared by an aqueous mini‐emulsion Suzuki‐Miyaura polymerization. Devices fabricated using the prototypical high performance donor–acceptor copolymer semiconductors poly(indacenodithiophene‐benzothiadiazole) (PIDTBT)^[^ ^28^ ^]^ and poly(diketopyrrolopyrrole thiophene‐benzothiadiazole) (PDPPTBT)^[^ ^29^ ^]^ are presented (**Figure** **2**a). The complete process from starting monomers to OFET devices involves aqueous processes, fewer steps, lower reaction temperatures, two orders of magnitude less VOCs and avoids the use of any halogenated solvents, during synthesis, purification or thin film processing.

**Figure 2:**
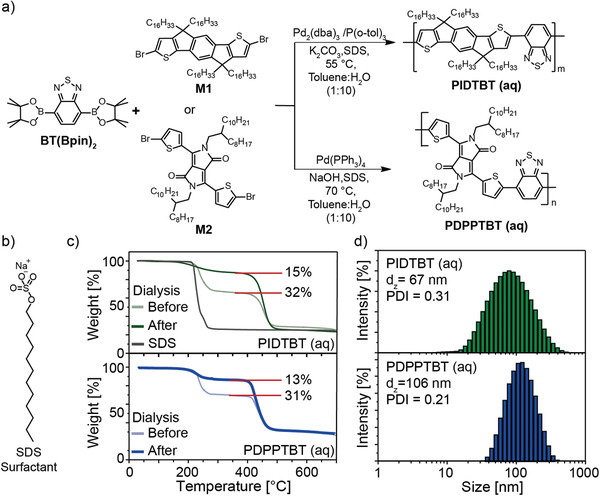
a) Reaction scheme for PIDTBT (aq) and PDPPTBT (aq) via mini‐emulsion Suzuki‐Miyaura polymerization; b) Molecular structure of SDS; NP dispersion characterization of PIDTBT (aq) (top green lines) and PDPPTBT (aq) (bottom blue lines); c) TGA to indicate removal of excess SDS (grey line) via dialysis, and d) DLS analysis to estimate the respective hydrodynamic particle size and dispersity distribution in water after dialysis.

The semiconducting polymers, PIDTBT and PDPPTBT, were prepared from borylated benzothiadiazole (BT(Bpin)_2_) with halogenated monomers of indacenodithiophene (M1) and diketopyrrolopyrrole‐thiophene (M2) respectively, under conventional and aqueous mini‐emulsion polymerization conditions. Detailed experimental procedures for the synthesis of both polymers are given in the methods sections and Table S1, Supporting Information gives an overview of the reaction conditions and results. Conventional polymerization methods were used to prepare material for control OFET devices processed using established literature procedures (see Figure S1, Supporting Information for the synthetic scheme).^[^ ^28^, ^29^ ^]^ Similar reagents were used for both conventional and mini‐emulsion reactions. The key difference between the two reactions was that sodium *n*‐dodecyl sulfate (SDS) and hexadecane were added to form the mini‐emulsion as a surfactant and a particle stabilizer, respectively. In addition, the volume ratio of the organic and aqueous phases was also different, under mini‐emulsion conditions a ratio of 1:10 toluene:water was employed whilst conventional conditions required a ratio of 4:1. The monomers, palladium catalyst and stabilizer were dissolved in the organic phase while the base and surfactant were dissolved in the aqueous phase. In the mini‐emulsion reaction the two phases were mixed using sonication to generate an emulsion of toluene droplets containing the respective components stabilized by the surfactant. This reaction was stirred at the specified temperature for 24 h. under an argon atmosphere. At the end of the reaction, the mixture was cooled to 40 °C and the toluene solvent removed by stirring for 2 h. under a continuous argon flow, this process generated an aqueous NP dispersion of PIDTBT (aq) and PDPPTBT (aq). To provide an additional comparison, the semiconducting polymers were isolated from the NP dispersion by precipitation in excess methanol; the solid was washed with methanol by centrifugation, filtered and dried before re‐dissolving in dichlorobenzene (DCB) to obtain solutions of PIDTBT (DCB) and PDPPTBT (DCB). The polymers synthesized using conventional methods were extracted in a similar manner and prepared as DCB solutions. It is important to note that soxhlet extraction using halogenated solvents (e.g., chloroform or chlorobenzene) to obtain uniform molecular weight distributions was not performed on precipitated polymers, and that this fractionation/purification is known to improve charge transport.^[^ ^30^ ^]^ Taking into consideration that polymers synthesized from mini‐emulsion reactions are directly processed into thin films, the exclusion of this step is to ensure a fair comparison of the effect of the molecular weight distribution of the polymers on charge transport in OFETs.

The number average molecular weight (*M_n_*) and dispersity (Đ) of the polymers isolated from the different reactions was estimated by GPC (see Table S1 and Figure S2, Supporting Information for GPC traces of the molecular weight distribution plots and values). Polymers synthesized using conventional methods were isolated with an *M_n_* of 20.9 kDa, *Đ* = 2.2 for PIDTBT at reaction temperatures (*T_r_*) of 90 °C, while PDPPTBT prepared at *T_r_* = 80 °C was isolated with an *M_n_* of 89.5 kDa and *Đ* = 1.7. The mini‐emulsion polymerization reactions yielded very similar polymers with an *M_n_* = 21.4 kDa, Đ = 2.3 for PIDTBT, and 81.6 kDa, *Đ* = 1.8 for PDPPTBT, but at lower *T_r_* of 55  and 70 °C respectively. Reactions conducted using conventional methods at these lower reaction temperatures gave low molecular weight oligomers (see Table S1, Supporting Information).

The aqueous NP dispersions were purified by a simple dialysis process against water to remove excess surfactant and water‐soluble impurities (see experimental procedure in Figure S3, Supporting Information for a detailed description). Purification by dialysis in water is in stark contrast to the standard purification procedures for semiconducting polymers that generally use soxhlet extraction with large quantities (100–200 mL for each wash at a similar reaction scale via conventional methods) of a variety of organic solvents (e.g., methanol, acetone, hexane and halogenated solvents). Thermogravimetric analysis (TGA) of the NP dispersions was used to quantify the surfactant removal by dialysis (see Figure 2c; green lines (top) for PIDTBT (aq) and blue lines (bottom) for PDPPTBT (aq)). SDS has a distinct decomposition temperature at an onset of 217 °C (Figure 2c, grey line) and TGA of the NP dispersions after preparation indicated that after 72 h. of dialysis, the surfactant concentration in the dispersions was effectively halved, from 32 and 31 wt% of SDS present for PIDTBT (aq) and PDPPTBT (aq) to 15 and 13 wt%, respectively (TGA data taken at each time interval are shown in Figure S4, Supporting Information). Dialysis for a further twelve hours did not lead to a further significant reduction in the amount of SDS present in the NP dispersion. Removal of excess surfactant is important as SDS is known to inhibit charge transport across the conduction channel of an OFET.^[^ ^21^, ^31^ ^]^ The concentration of polymer in dispersions was estimated to be 0.9 and 1.0 wt% for PIDTBT (aq) and PDPPTBT (aq), respectively, based on the measured weight loss of the polymer. Dynamic light scattering (DLS) analysis after dialysis (Figure 2d) showed that the NPs have a hydrodynamic particle size (nm)/polydispersity index (*d*_z_/PDI) of 67/0.31 for PIDTBT (aq) (top histogram in green) and 106/0.21 for PDPPBT (aq) (bottom histogram in blue). Importantly, the size and dispersity of the NP dispersions for both polymers remained relatively unchanged throughout the dialysis procedure (see Figure S5, Supporting Information for d_z_/PDI values). Furthermore, the NP dispersions were stable over a period of three months under ambient conditions when stored in the dark (see Figure S6, Supporting Information for DLS and UV‐Vis data). There was no change in the d_z_/PDI values of PIDTBT (aq) while a small increase in d_z_/PDI values for PDPPTBT(aq) to 167/0.27 was observed. No significant change in the optical absorption spectrum for both dispersions was observed.

The NP dispersions were deposited on glass substrates by spin coating and the morphology of the NPs and thin films investigated by atomic force microscopy (AFM). Spin coating of dilute NP dispersions (0.1 wt%) gave isolated NP aggregates on the glass surface. The particle size of the polymers in these granular aggregates was estimated to be 55 ± 15 nm for PIDTBT and 90 ± 23 nm for PDPPTBT (see Figure S7, Supporting Information). These values agreed well with the values determined by DLS (see Figure 2d). Spin‐coating of higher concentration NP dispersions obtained by dialysis gave thin films of NP with a uniform morphology of tightly packed granular particles as shown in **Figure** **3**a,b for PIDTBT (aq) and PDPPTBT (aq), respectively. The deposition of a continuous NP morphology in thin films is crucial for device performance.^[^ ^21a^ ^]^ Thermal annealing of the as‐deposited PIDTBT (aq) films at 150 °C for 30 min, resulted in the coalescence of the particles to form a continuous amorphous film (see Figure 3c). In contrast, annealing of the PDPPTBT (aq) film at 150 °C for 30 min resulted in only minor changes, with a decrease in RMS roughness from 2.4 to 0.5 nm (see Figure 3d). Thin films of the polymers were also deposited by spin coating from DCB solution (Figure S8, Supporting Information). PIDTBT (DCB) showed an amorphous morphology, similar to that observed for the annealed, aqueous‐processed thin films, while PDPPTBT (DCB) showed a fibrillary texture, after annealing at 150 °C. These observations are consistent with thin films of these materials deposited from organic solvents that have been previously reported.^[^ ^29^ ^]^ The feasibility of using these aqueous NP dispersions to deliver functional semiconducting thin films was demonstrated by the fabrication of top‐gated OFETs on glass substrates, using poly (methyl methacrylate) (PMMA) as the gate dielectric. For a detailed description of the fabrication steps, refer to Figure S9, Supporting Information and the experimental procedures section. The charge carrier mobilities (µ) in OFETs processed from either aqueous dispersion or DCB solution were calculated in the saturation regime of the transfer curves taken from 12 devices and the data is presented in Figure 3e. The transfer and output characteristics are shown in the Figures S10 and S11, Supporting Information, for PIDTBT and Figures S12 and S13, Supporting Information, for PDPPTBT and the extracted parameters are collected in Table S2, Supporting Information.

**Figure 3:**
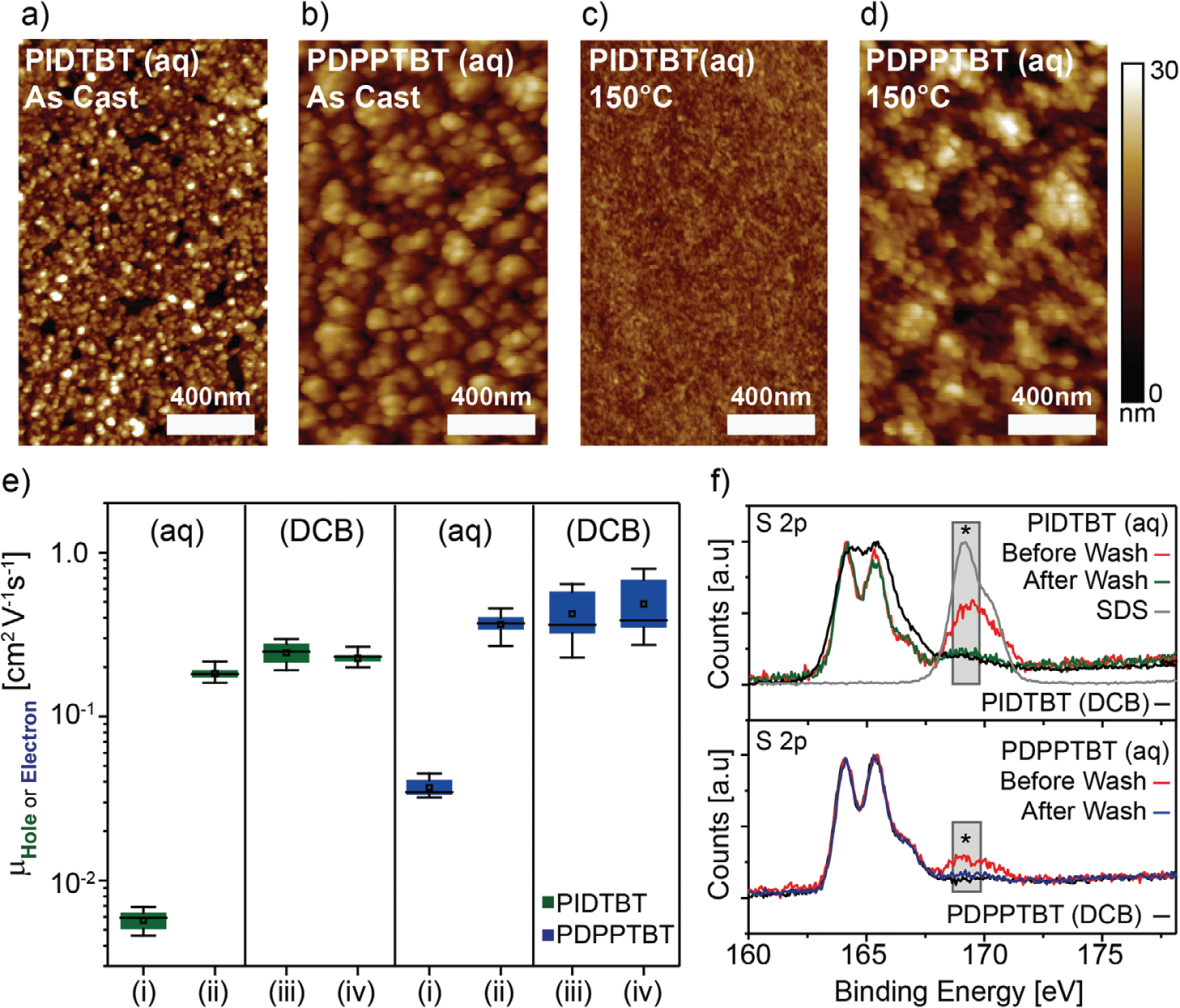
Atomic force microscopy thin‐film images of aqueous‐processed NP dispersions of PIDTBT (aq) and PDPPTBT (aq): a,b) as‐cast, c,d) annealed films at 150 °C for 30 min. Box and whisker plot e) showing a summary of saturation mobility values (*µ*) taken from 12 devices of OFETs processed from NP (aq) and DCB dispersions of PIDTBT (Green) and PDPPTBT (Blue): (i) before and (ii) after surfactant wash, and DCB dispersions of polymers synthesized from (iii) mini‐emulsion and (iv) conventional methods. Transfer characteristics were measured in the saturation regime under an applied bias of *V*_DS_ = *V*_GS_ = −80 V for p‐type behavior, and *V*_DS_ = 40 V and *V*_GS_ = 60 V for n‐type behavior (see Figure S10, Supporting Information for full transfer and output curves and Table S2, Supporting Information for an overview of transistor performance including literature reported values of equivalent OFET devices. X‐ray photoelectron spectroscopy analysis f) of aqueous‐processed thin‐film before (red lines) and after the post‐washing process (green line for PIDTBT and blue line for PDPPTBT). Grey box with (:) indicates the region of the S 2p sulfate peak and including DCB‐processed semiconducting polymer thin films (black line).

Devices fabricated using PIDTBT showed p‐type hole transport (μ_h_) while PDPPTBT showed n‐type electron transport (µ_e_). Transistors from as‐cast aqueous‐processed thin films showed poor performance with PIDTBT (aq) and PDPPTBT (aq) achieving μ_h_ = 2.2 × 10^−4^ ± 10^−4^ cm^2^ V^−1^ S^−1^, and *µ*_e_ = 1 × 10^−3^ ± 10^−3^ cm^2^ V^−1^ S^−1^, respectively. It appears that the surfactant present in these as‐cast thin films acts as charge trapping sites as discussed earlier, limiting the device performance. Thermal annealing of these films at 150 °C did not lead to any significant improvement in device performance (See Table S2, Supporting Information). To effectively remove any surfactant remaining in the thin film, a post‐washing process was used. Ethanol was drop‐cast on the thin‐film, left for 60 s and then removed by spinning the film at 6000 rpm. The films were then annealed at 100 °C for 5 min to evaporate any remaining solvent. This post‐washing process resulted in a significant improvement in the transistor performance with mobilities increasing to μ_h_ = 0.18 ± 0.02 cm^2^ V^−1^ for PIDTBT (aq) and µ_e_ = 0.36 ± 0.05 cm^2^ V^−1^ S^−1^ for PDPPTBT (aq). The effectiveness of the post‐washing step was investigated by X‐ray photoelectron spectroscopy (XPS) (see Figure 3f). Observation of a S 2p peak at 168.5 eV (see (:) in the grey region) is assigned to the presence of the sulphate group on the SDS molecule in the thin film. It is clear that the intensity of the sulphate peak observed for both aqueous‐processed thin films was significantly reduced after the post‐washing step and after washing showed a similar intensity to that observed for the DCB‐processed thin films. This implies that a simple washing step is sufficient to remove the surfactant from the films.

OFETs have been previously reported that were fabricated from an aqueous dispersion of semiconducting polymer NPs prepared by emulsification of an organic solution of a pre‐polymerized OSC and SDS as a surfactant (see Figure 1b). A brief summary of these previous reports listing the type of polymer, the concentration of polymer and the amount of SDS used to generate the dispersion is collected in Table S3, Supporting Information. In these approaches, the polymer was synthesized and purified by conventional solvent‐based processes, and for SDS based emulsions the transistor performances did not improve significantly on washing of the thin films.^[^ ^21^, ^31^ ^]^ A harsh thermal treatment at 270 °C was required to eliminate the surfactant from the thin film and these processing conditions are not compatible with the majority of flexible substrates.^[^ ^21d^ ^]^ In these previous reports, a high concentration of SDS in the aqueous phase (1 to 2 wt%), and a low concentrations of the semiconducting polymer (≤0.5 wt%) in a halogenated solvent (chloroform) was required to deliver stable aqueous dispersions. The approach presented in this contribution utilizes a lower concentration of SDS (0.75 wt%) to achieve a stable dispersion in combination with a non‐halogenated solvent, i.e., toluene. In addition, the high solubility of the monomers in this solvent facilitates the polymerization and generation of stable NP dispersions of PIDTBT (aq) and PDPPTBT (aq) at higher concentrations of the polymer (≥0.9 wt%) with respect to the amount of water. The amount of SDS was further reduced by dialysis against water to levels that could be removed from the thin film using a simple spin‐washing step, leading to improved transistor performance. XPS of the thin films taken at a wide range of binding energy showed no detectable palladium or phosphorus signals associated with the palladium catalyst before or after washing (see Figure S14, Supporting Information). In addition there were no observable transition peaks in the TGA trace that could be assigned to the presence of the particle stabilizer, hexadecane (boiling point = 287 °C, Figure 2c). Although these observations do not confirm that these components have been removed, they suggest that the low concentrations of hexadecane (0.35 mol%) and palladium catalyst (<2 mol%) used in the synthesis do not influence the charge transport in the conduction channel of polymer during transistor operation.

To provide a realistic comparison of the performance of the OFETs processed from water, analogous devices were fabricated using DCB solutions of the respective polymers synthesized by conventional and mini‐emulsion methods (see Figure 3e). Transistors processed from DCB showed similar performance for mini‐emulsion and conventionally polymerized material, where PIDTBT (DCB) achieved *μ*_h_ = 0.24 ± 0.04 cm^2^ V^−1^ and *μ*_h_ = 0.23 ± 0.03 cm^2^ V^−1^, while PDPPTBT (DCB) also showed similar mobilities *μ*_e_ = 0.43 ± 0.10 cm^2^ V^−1^ S^−1^ and *µ*_e_ = 0.48 ± 0.20 cm^2^ V^−1^ S^−1^ respectively. Most promisingly, the performance of the aqueous‐processed devices for both PIDTBT and PDPPTBT were comparable to those of the DCB‐processed devices. Furthermore, reported values for the OFET performance of these polymers in equivalent top‐gated device architectures also showed comparable mobilities for both polymers^[^ ^32^ ^]^ (SeeTable S2, Supporting Information for μ values).

In summary, we report an efficient synthesis‐to‐device process for OSC‐based transistors using more environmentally benign aqueous processes for all key steps. The NP dispersions based on p‐type PIDTBT (aq) and n‐type PDPPTBT (aq) semiconducting polymers were successfully prepared by mini‐emulsion Suzuki‐Miyaura polymerization , yielding quantitative conversion and comparable molecular weights to conventional polymerization methods in organic solvents, conducted at higher temperatures. Soxhlet extraction with halogenated solvents was avoided, and only water was used to purify the NP dispersions. Stable, uniform NP dispersions at higher concentrations of the polymer in water were obtained with less surfactants due to the use of readily soluble conjugated monomers at the start of the mini‐emulsion polymerization process. This enabled the deposition of these NP dispersions to form homogenous and continuous thin film morphologies of tightly packed NPs that were crucial for continuous charge transporting pathways during transistor operation. Effective removal of the remaining surfactant was possible using a simple spin‐washing step after thin film formation. Encouragingly, aqueous‐processed transistors for both polymers showed comparable charge transport mobilities to devices processed using halogenated solvents. Ultimately, the work described exploits aqueous processes as the medium for polymerization, purification and solution‐processing of the OSC into thin films. We estimate that there is a >99% reduction in the use of organic solvents using the aqueous NP dispersions approach compared to a conventional synthesis‐to‐device approach of the same scale (See Table S4, Supporting Information for calculations). This addresses the pervasive use of large amounts of VOCs, and more importantly avoids the use of toxic halogenated solvents during the synthesis‐to‐device process. Furthermore, current strategies that seek to address the environmental challenges associated with OSC synthesis and processing only focus on the individual steps in the synthesis to device process, such as developing alternative synthetic methodologies (e.g., atom‐economical direct arylation reactions),^[^ ^33^ ^]^ utilizing green solvents for solution‐processing of thin films,^[^ ^9^, ^13^ ^]^ or employing biodegradable and sustainable components of the OSC or device substrates.^[^ ^10^, ^34^ ^]^ These approaches are important and have been successful in addressing each individual environmental challenge and achieving high performance OSC devices, but do not provide a holistic pathway for a complete synthesis‐to‐device process. The presented streamlined synthesis‐to‐device process represents a viable template for the development of other types of OSCs and the fabrication of relevant devices such as organic electrochemical transistors, organic photovoltaics and organic light‐emitting diodes.

## Experimental Section

The experimental procedures and materials used are included in the Supporting Information.

## Conflict of Interest

The authors declare no conflict of interest.

## Supporting information

Supporting Information
