# Supplementary material for: Organic Semiconductors Processed from Synthesis‐to‐Device in Water
Source: Adv Sci (Weinh). 2020 Sep 21;7(21):2002010. doi: 10.1002/advs.202002010 (PMC7610335; doi:10.1002/advs.202002010)
Supplement: Supplementary file 1 — Supporting Information [file ADVS-7-2002010-s001.pdf]

© 2020 Wiley-VCH GmbH

ADVANCED SCIENCE
Open Access

Supporting Information

for *Adv. Sci.,* DOI: 10.1002/advs.202002010

Organic Semiconductors Processed from Synthesis-to-Device in Water

Aiman Rahmanudin, Raymundo Marcial-Hernandez, Adibah Zamhuri, Alex S. Walton, Daniel J. Tate, Raja U. Khan, Suphaluk Aphichatpanichakul, A. B. Foster, S. Broll, Michael L. Turner**

© 2020 Wiley-VCH GmbH

Supporting Information

Organic Semiconductors Processed from Synthesis-to-Device in Water

Aiman Rahmanudin, Raymundo Marcial-Hernandez, Adibah Zamhuri, Alex S. Walton, Daniel J. Tate, Raja U. Khan, Suphaluk Aphichatpanichakul, A. B. Foster, S. Broll, Michael L. Turner**

Supporting figures mentioned in the main text.

Table S1. Summary of results for the synthesis of PIDTBT and PDPPTBT

Entry	OSC	Method [a]	Monomer Concentration [mmol/mL]	Volume Ratio Organic:Water [mL]	Base	T_Δ [°C]	M_n [kDa] [b]	Đ [b]	Yield [%]
**1a**	PIDTBT	Conventional	0.07	4:1	Na_2CO_3	90	20.9	2.2	83
**1b**						55	1.2	1.21	32[c]
**1c**		Mini-emulsion	0.20	1:10		90	17.0	1.9	82
**1d**						55	21.4	2.3	79
**2a**	PDPPTBT	Conventional	0.06	4:1	K_2CO_3	80	89.5	1.74	85
**2b**						70	3.74	1.51	67
**2c**					NaOH	80	4.54	1.53	56
**2d**		Mini-emulsion	0.15	1:10	K_2CO_3	80	71.5	1.64	81
**2e**					NaOH	80	81.8	1.99	86
**2f**						70	81.6	1.78	82

[a] Detailed synthetic procedures are described below in the method section.
[b] For M_n and Đ values, analytical gel permeation chromatography measurements were performed @ 160 °C in trichlorobenzene of the polymers that were precipitated in methanol. M_n and Đ were calculated using PS as reference. Molecular weight plots for each polymers at different conditions are shown in Figure S2 below.
[c] Low yield is due to low polymer conversion with mainly unreacted starting monomers.

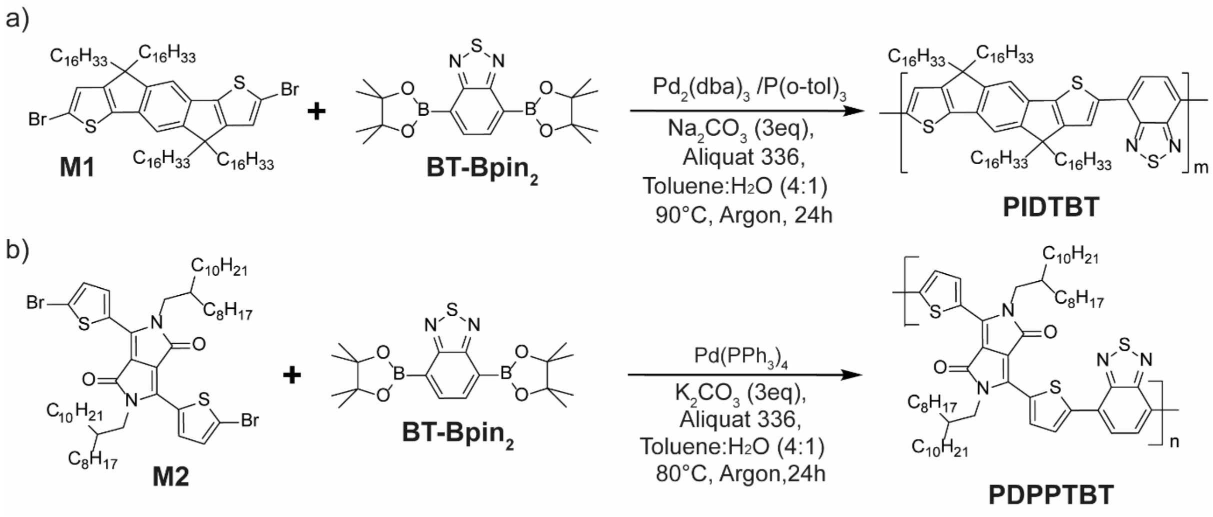

Figure S1. Reaction scheme of the polymer semiconductor synthesised from conventional polymerization method of a) PIDTBT and b) PDPPTBT.

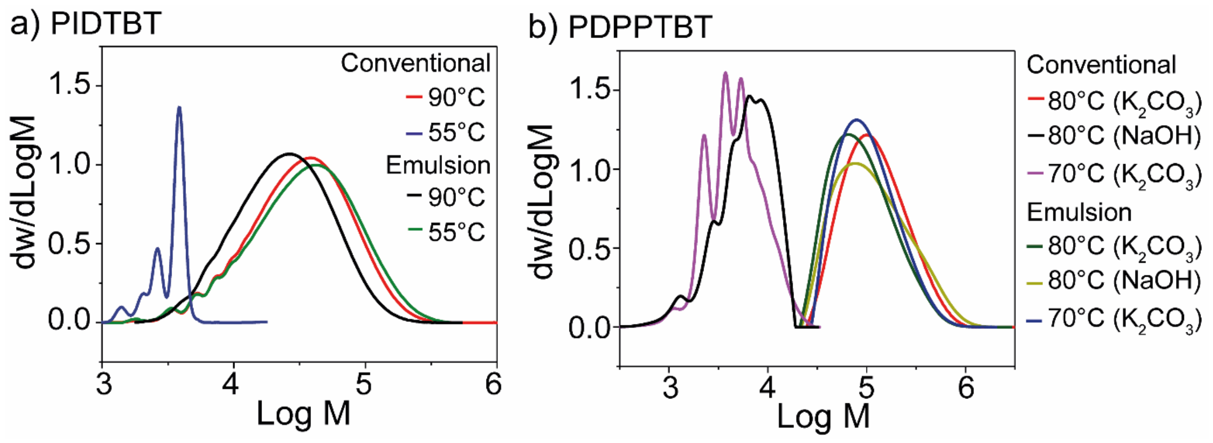

Figure S2. Analytical gel-permeation chromatography (GPC) traces of a) DPPTBT and b) IDTBT synthesized from mini-emulsion and conventional polymerization conditions.

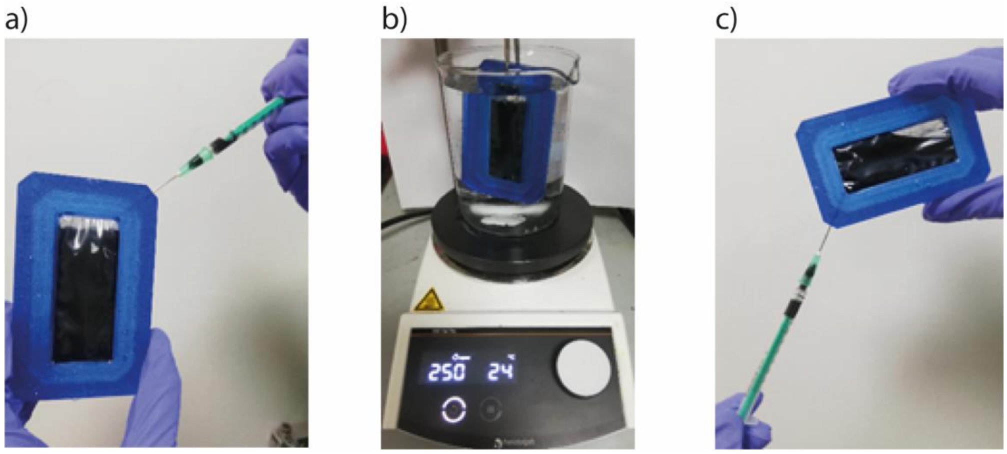

Figure S3. Images of dialysis purification steps: a) The NP dispersions taken from the Schlenk tube after each reaction was injected into a Slide-A-Lyzer Dialysis Cassettes with a 2000 molecular weight cut-off (MWCO) permeation membrane; b) the cassette is completely immersed in deionised (DI) water and left to stir over a period of 72 hrs. DI water was replaced every 12 hours; c) Approximately 200 µL of the dispersionis taken out at every time interval to characterise the level of surfactant removed via TGA, and DLS measurements for stability of the NP dispersions. At the end of the dialysis process, the remaining NP dispersion was removed from the cassette, and filtered through a 1µm glass Acrodisc filter before processing into thin-films for OFETs.

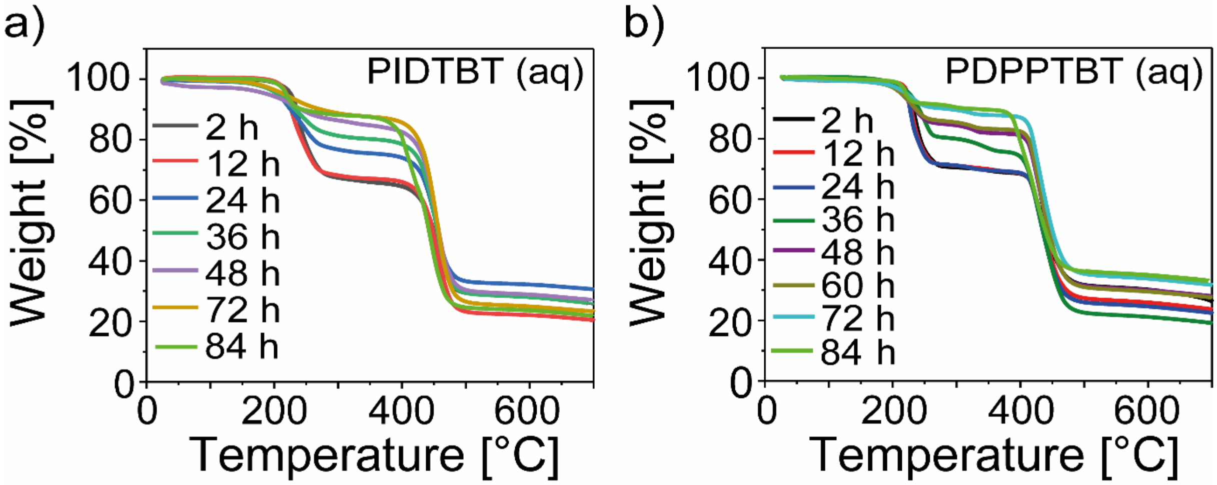

Figure S4. TGA of NP dispersions taken at 12 hrs intervals during dialysis purification to characterize surfactant removal: a) PIDTBT (aq) and b) PDPPTBT (aq).

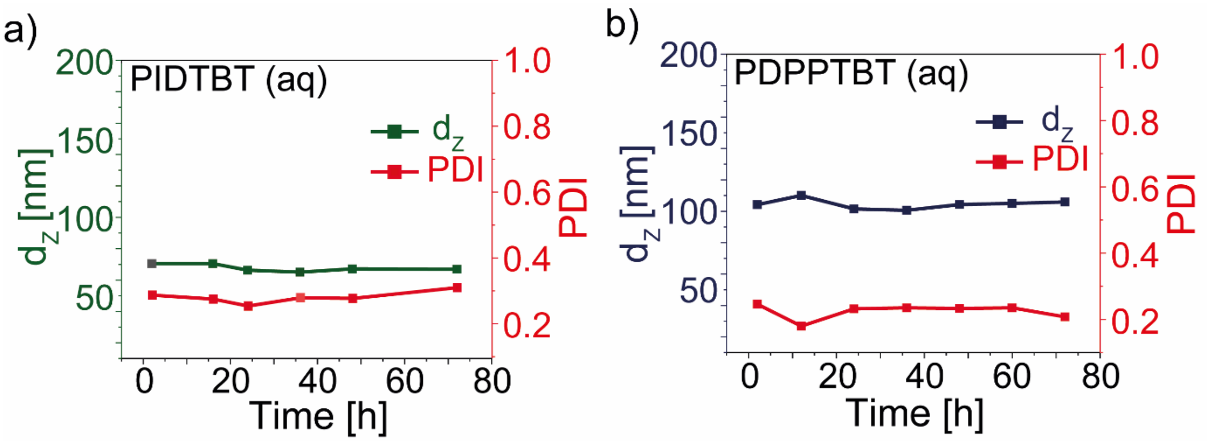

Figure S5. DLS analysis of the NP dispersions throughout the dialysis process: a) PIDTBT (aq) and b) PDPPTBT (aq).

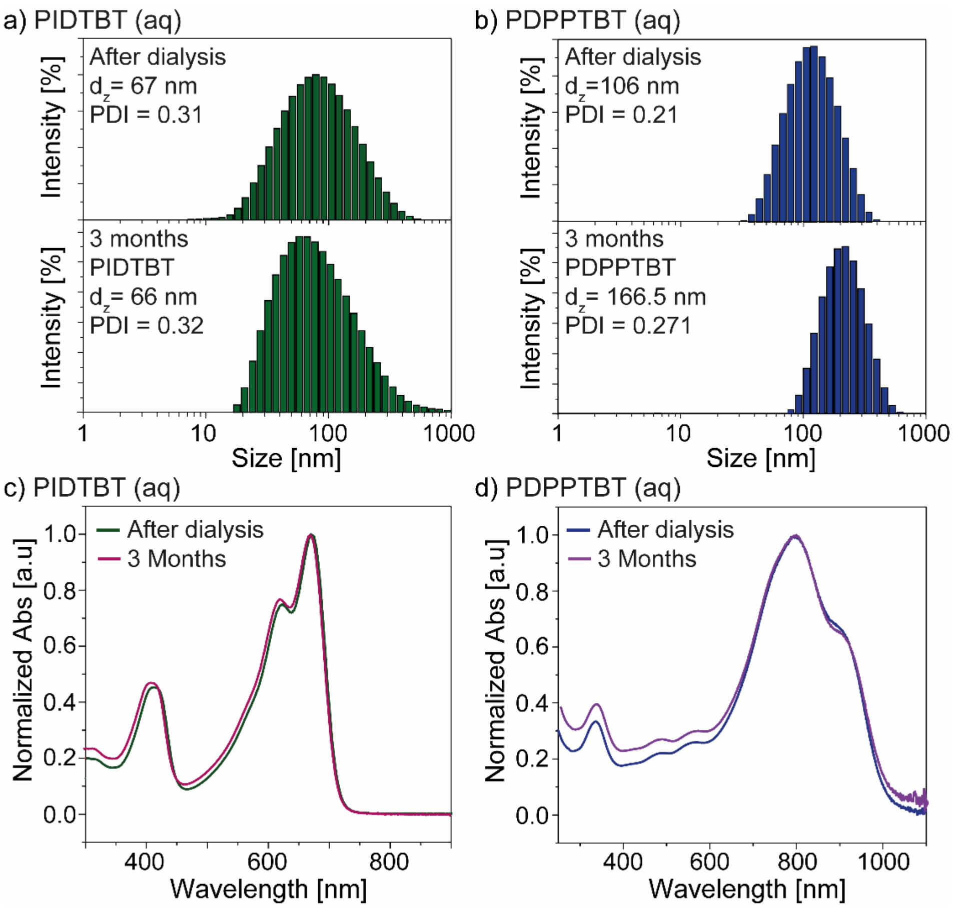

Figure S6. Stability of PIDTBT (aq) and PDPPTBT (aq) NP dispersions kept under ambient conditions in the dark over a period of 3 months: a-b) DLS analysis of the average hydrodynamic particle size (nm)/polydispersity index (d_z/PDI), and c-d) optical absorption spectra before and after 3 months.

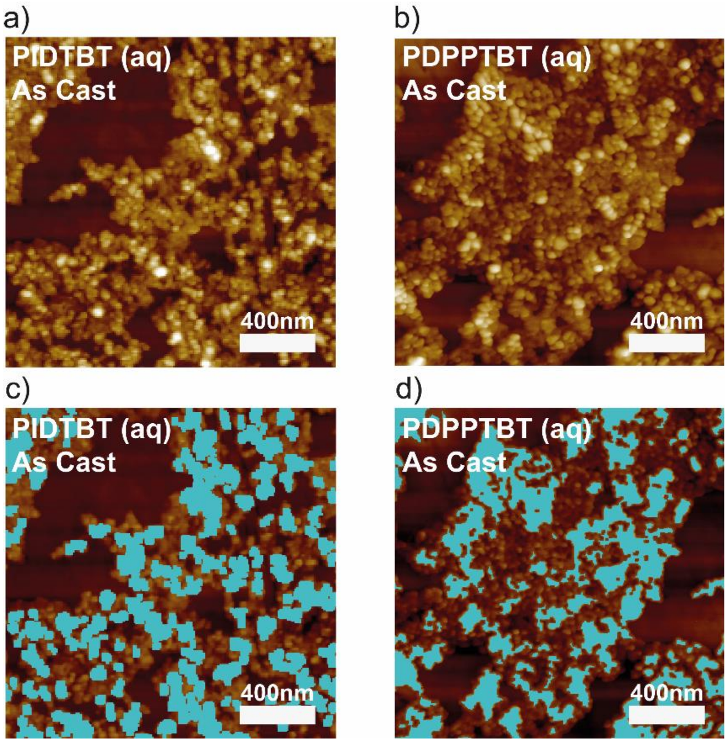

Figure S7. Atomic force microscopy (AFM) images of spin coated thin-films of a) PIDTBT (aq) and b) PDPPTBT (aq) at 0.1 wt% NP dispersionconcentration. The size of the NPs was estimated using the Bruker NanoScope Particle Analysis software, where the light blue markings are the particles detected: c) PIDTBT - Total count = 145; 55 ± 15 nm d) PDPPTBT - Total count = 84; 90 ± 23 nm.

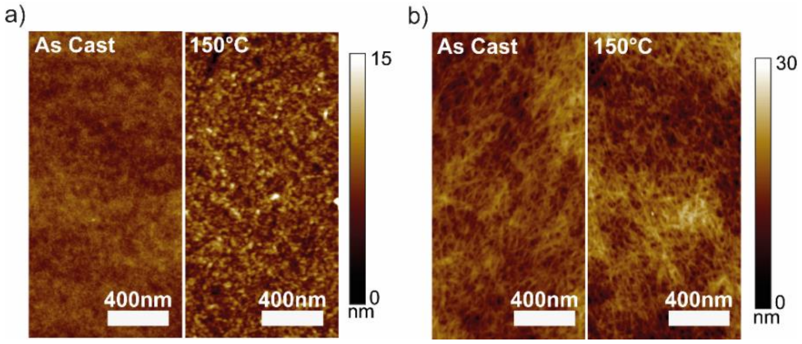

Figure S8. Atomic force microscopy (AFM) thin-film images for OSCs polymerised from conventional methods deposited from DCB dispersions: a) PIDTBT (DCB), b) PDPPTBT (DCB).

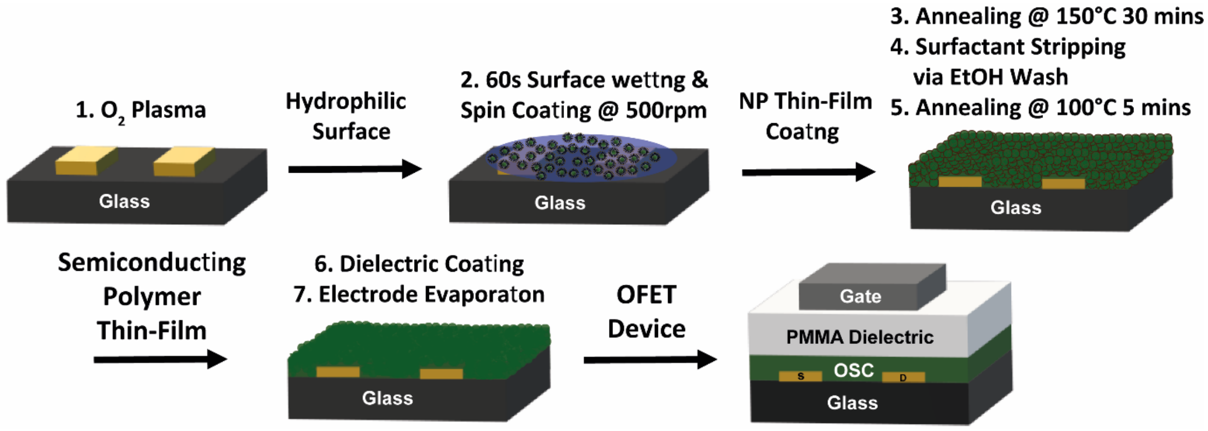

Figure S9. Schematic of the top-gated OFET fabrication procedure for processing of the NP (aq) dispersions. Refer to methods section for further details of the device fabrication process.

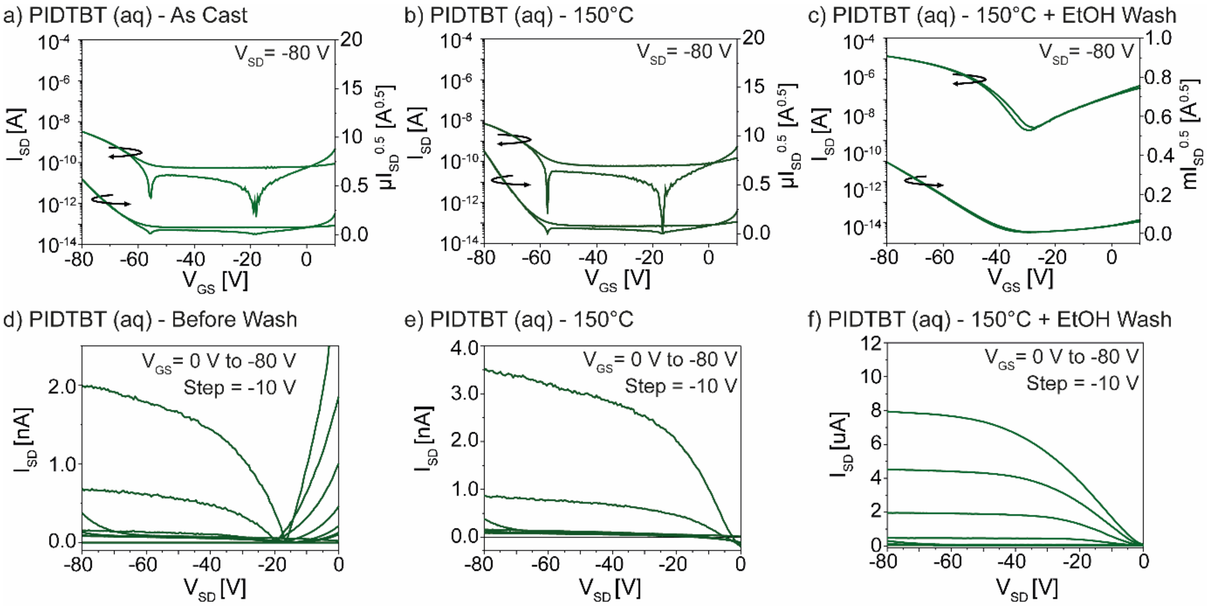

Figure S10. Representative transfer (a-c) and output (d-f) characteristics of aqueous-processed devices from PIDTBT (aq).

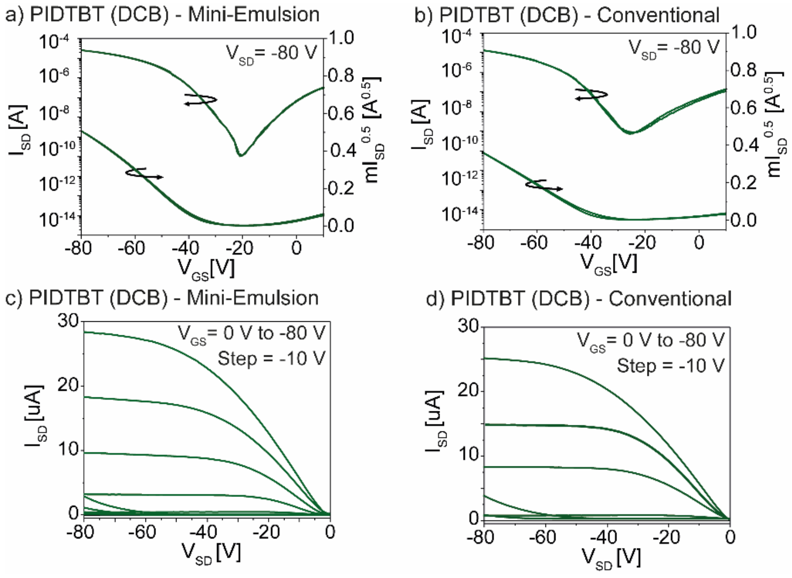

Figure S11. Representative transfer (a-b) and output (c-d) characteristics of DCB-processed devices from PIDTBT (DCB).

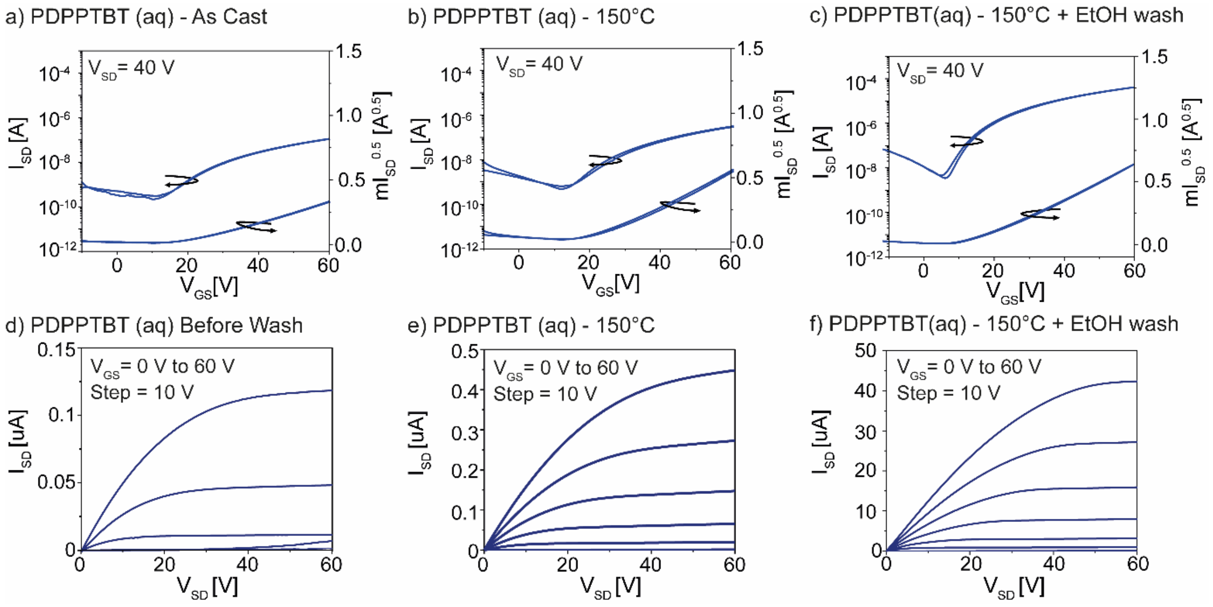

Figure S12. Representative transfer (a-c) and output (d-f) characteristics of aqueous-processed devices from PDPPTBT (aq).

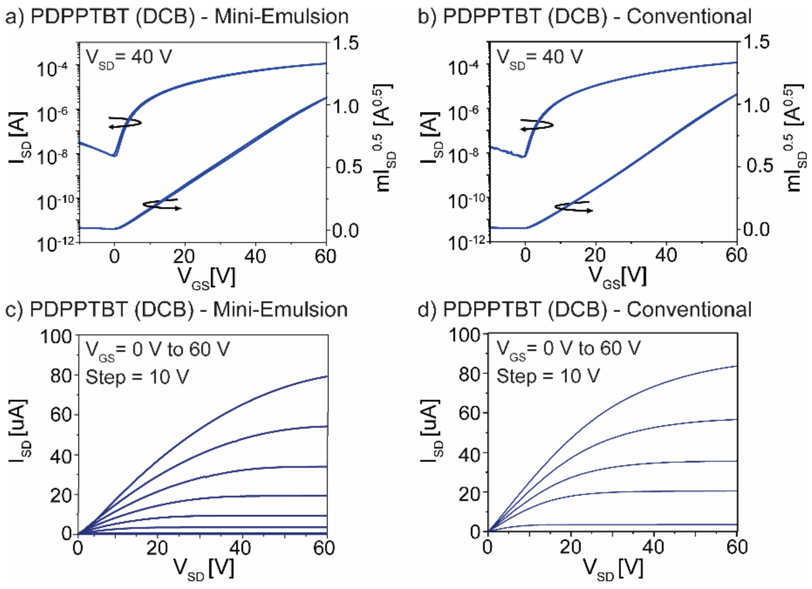

Figure S13. Representative transfer (a-b) and output (c-d) characteristics of DCB-processed devices from PDPPTBT (DCB).

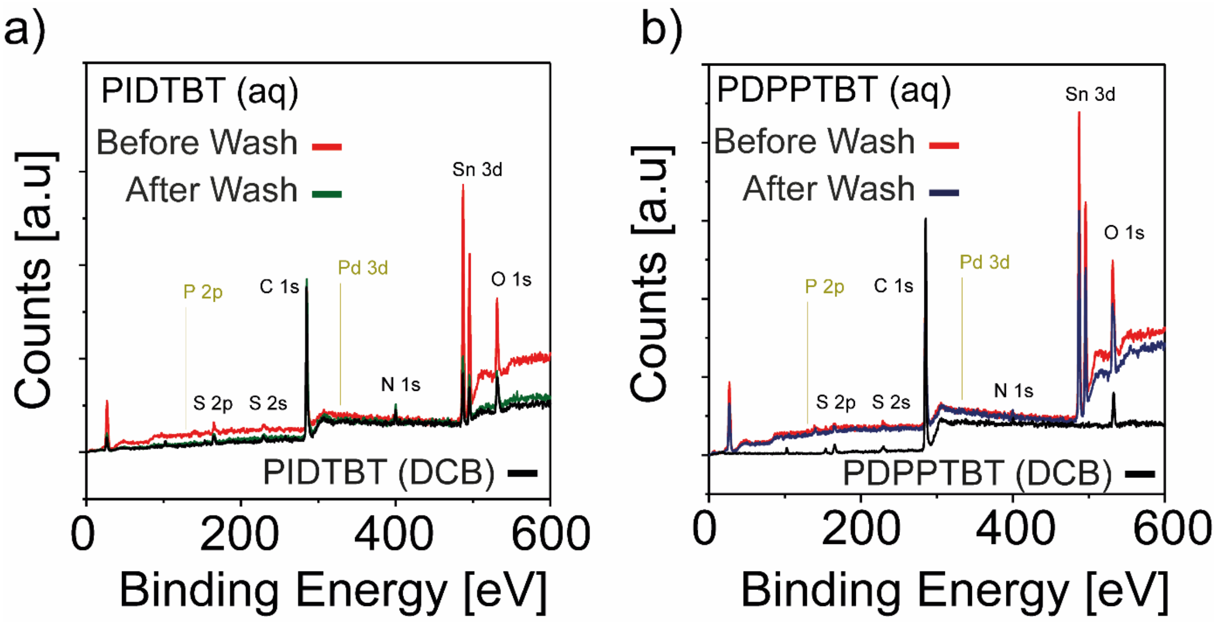

Figure S14. XPS scans at a wider range of binding energy (eV) for a) PIDTBT and b) PDPPTBT thin films: Yellow lines indicate the binding energy associated with the Palladium catalyst and Phosphine ligands. Sn 3d peaks are related to the ITO substrates used for the experiment.

Table S2. Overview of OFET performance

Entry	OSC	Method	Processing	T_a [°C]	Purification / Washing step	$\mu_{(hole\ or\ electron)}$ [$cm^2V^{-1}s^{-1}$]	V_{th} [V]	I_{on}/I_{off}
Ref [a]	PIDTBT	Conventional	DCB	150		0. 17 ± 0.04	-25 ± 1	2.7 x 10^2
1a					MeOH Precip.	0. 23 ± 0..03	-29 ± 4	1.9 x 10^2
1b		Mini-Emulsion			MeOH Precip.	0.24 ± 0.04	-33 ± 3	5.7 x 10^2
1c			Water	As Cast	Dialysis	2.2 x 10^{-4} ± 1.2 x 10^{-4}	-63 ± 12	1.2 x 10^3
1d				150	Dialysis	0.006 ± 8.2 x 10^{-4}	-61 ± 2	1.1 x 10^2
1e				150	Dialysis + EtOH	0.18 ± 0.02	-38 ± 5	3.2 x 10^2
Ref [b]	PDPPTBT	Conventional	DCB	150		0.49 ± 0.1		
2a					MeOH Precip.	0.48 ± 0.2	1.2 ± 1	1.7 x 10^4
2b		Mini-Emulsion			MeOH Precip.	0.43 ± 0.1	5.6 ± 1	1.3 x 10^4
2c			Water	As Cast	Dialysis	0.0014 ± 0.002	33 ± 3	4.7 x 10^2
2d				150	Dialysis	0.036 ± 0.005	25 ± 4	6.5 x 10^2
2e				150	Dialysis + EtOH	0.36 ± 0.05	19 ± 2	7.8 x 10^3

[a] H. Opoku., *et.al. J. Mater. Chem. C.* **2018,** 6, 661-667.
[b] Z. Ni., *et.al. Nature Chem.* **2019,** 11, 271-277

Table S3. Overview of OFETs processed from aqueous NP dispersions prepared from the emulsification of pre-polymerised OSCs using SDS as surfactant

Ref	OSC	Organic: Aqueous Phase ratio [Chloroform:Water]	Concentration OSC (organic) [mg mL^{-1}]	Concentration SDS (aqueous) [mg mL^{-1}]	Surfactant Removal	Deposition	$\mu_{(hole\ or\ electron)}$ [$cm^2V^{-1}s^{-1}$]
a	PNDI-TVT	1:3	2.5	20	Ethanol dipping for 3 mins	Spray	μ_e = 1 x 10^{-3} ± 0.00027
b	DPP-SVS	1:1	2.0	10	Repeated precipitation and re-dispersion via centrifugation and Ethanol dipping for 3 mins	Drop	μ_h = 3 x 10^{-3} ± 0.001
c	P3HT	1:2.8	30	15	Dialysis	Spin	-
d	P3HT	1:4	5	1mM or 16mM	Repeated precipitation and re-dispersion via centrifugation	Spray	Time of Flight μ_h = 4 x 10^{-4}
e	PBTTT	2:3	2.5	16.7	Repeated precipitation and re-dispersion via centrifugation/ Dipping in water/ 200 or 270 °C for 5s	Spin	3.2 x 10^{-2}
This work	PIDTBT	Toluene:Water 1:10	9.0	7.5	Dialysis and Ethanol Spin wash	Spin	μ_h = 0.17 ± 0.03
	PDPPTBT	Toluene:Water 1:10	10.0				μ_e = 0.36 ± 0.1

[a] J. Cho., *et.al. Energy Environ. Sci.* **2017,** 10, 2324-2333.
[b] J. Cho., et.al. *Adv. Mater,* **2015,** *27*, 5587-5592.
[c] D. Darwis., *et.al. J. Colloid Interface Sci.* **2013,** 401, 65-69.
[d] M. Bag., *et.al. Adv. Mater.* **2013,** 25, 6411-6415.
[e] J. Cho., *et.al. Chem. Eng. J.* **2016,** 286, 122-127.

Table S4. Comparison of estimated organic solvent used at each key step between conventional and NP dispersion synthesis-to-device processes

Method	Synthesis		Purification		Processing		Total volume of organic solvent used [mL]
	Organic [mL]	Water [mL]	Organic[a] [mL]	Water[b] [mL]	Organic [mL]	Water [mL]	
Conventional	4 (Toluene)	1	100 x 3 (Methanol, Hexane, and Chlorobenzene or Chloroform)	-	0.05 per spin coat (Chlorobenzene or Chloroform)	-	305.05 (99.7%)
Mini-emulsion Polymerization	1 (Toluene)	10	0	100 (x 7) + 10	0.25 Surfactant washing (Ethanol)	0.05 per spin coat	1.25 (0.3%)

[a] Volume of organic solvents are estimated for soxhlet extraction based on the same scale of reaction as described in the methods section in the main text

[b] 100mL required to immerse the dialysis cassete containing 10 mL of the NP dispersion for purification from dialysis. This volume of water is replenished at least 7 times over a period of 72hrs in accordance to Figure S4.

Experimental Methods

Materials: All reagents were purchased from Sigma-Aldrich, Alfa Aesar, Acros and were used without further purification unless indicated. Solvents were purchased from Sigma-Aldrich or Alfa Aesar. Deionised (DI) water was used for dialysis. Aqueous solution used for the polymerization reactions were prepared using DI water that was bubbled with Argon gas for 72 hours prior to use. Monomer synthesis of M1 and M2 were achieved according to literature procedures.[32a] 2,1,3-Benzothiadiazole-4,7-bis(boronic acid pinacol ester) ($BT(Bpin)_2$) was purchased from Ossila Ltd and used without further purification.

Synthesis Characterization. ^1H and ^{13}C NMR spectroscopy experiments were recorded on Bruker Avance 400 spectrometer. Chemical shifts are reported in parts per million (ppm) relative to the residual solvent (for instance $CHCl_3$ in $CDCl_3$ δ H = 7.26 ppm, δ C =77.36 ppm). The multiplicity of the signals is reported using the following abbreviations: s=singlet, d=doublet, t=triplet, dd=doublet of doublet, ddd=doublet of doublet of doublets, ddt= doublet of doublet of triplets, m=multiplet. Gel permeation chromatography (GPC) was performed on Agilent 1260 Infinity II system at 160 °C using 1,3,5-trichlorobenzene as an eluent. The average molecular weight in number (M_n), in weight (M_w), and weight average dispersity Đ (M_w/M_n) was determined using narrow weight average dispersity (Đ < 1.10) polystyrene (PS) standard. GPC samples were prepared by precipitation of the crude product from the respective reactions in excess methanol. The solid was isolated by centrifugation and the supernatant decanted several times before drying under vacuum at 40 °C.

NP Dispersion Characterization. Dynamic Light Scattering (DLS) measurements using a Malvern Zetasizer Nano ZS instrument were used to determine the particle size of the NPs dispersed in water. An aliquot (5 µL) of the NP dispersion was diluted into a 1 ml volume of DI water before measuring each sample at 25°C. The DLS results are quoted from an average

of three measurements. Thermogravometric analysis (TGA) measurements were performed on a Discovery SDT650 TGA from TA instruments. The samples were measured at 10 °C/min under a N_2 atmosphere in an alumina pan from 30°C to 800 °C. Samples were prepared by sequential addition of 20 µL x 10 (Total = 200 µL) of the respective NP dispersions into the alumina pans left at 80°C to allow the solvent (water) to evaporate to obtain a minimum mass of approximately 2 mg. The concentration of each respective NP (aq) dispersion was estimated from the measured wt% loss of the polymer with respect to the amount of water. For PIDTBT (aq) and PDPPTBT (aq), 1.8 mg (90 %) and and 1.9 mg (95 %) was measured to give an estimated NP (aq) concentration of 0.9 wt% and 1.0 wt%, respectively.

General procedure for mini-emulsion polymerization synthesis. Firstly, SDS (75 mg) and the relevant base (3 molar equivalents) were dissolved in 10 ml of DI water in an argon purged Schlenk tube. The aqueous solution was stirred and bubbled with argon gas for a further 30 minutes to ensure complete dissolution of the reagents. The organic phase was prepared in a separate argon purged Schlenk tube by first adding the relevant catalyst, ligand, particle stabilizer (hexadecane), and monomer M1 or M2 and $BT(Bpin)_2$ at the corresponding stoichiometric amounts for each polymer reaction. The reagents were then dissolved in 1 mL of anhydrous toluene and subsequently bubbled with argon gas for 5 minutes. Next, the organic solution was injected into the Schlenk tube containing the aqueous solution and this was transferred into an ice bath. The sonicator tip was then submerged into the reaction mixture under an argon purge, and sonicated for a total of 4 minutes (2 x 2 minutes) using a Sonics VCX-750 Vibra Cell ultrasonicator at 22% amplitude and a 6.4 mm sonicator tip. The Schlenk tube was then immediately sealed and placed into a heated oil bath at the corresponding reaction temperature and stirred for 24 h. The reaction was cooled to 40 °C, left unsealed under argon flow for 2-3 h to evaporate the toluene solvent to give the NP dispersion. This dispersion was then injected into a Thermo Scientific Slide-A-Lyzer dialysis cassette (2K MWCO), immersed

in DI water and left to stir over a period of 72 hours. The DI water was replaced every 12 hours. After dialysis, the volume of the solution had the tendency to increase slightly to approximately 12mL. The NP dispersions were left to stir in a Schlenk tube under an argon flow to evaporate the excess water until the total volume returned to 10mL. The resulting NP dispersion was used for further characterization and processing into thin-films. **PIDTBT (aq).** The synthesis was performed according to the general procedure described above. K_2CO_3 (31.4 mg, 0.22 mmnol) and SDS (75 mg) were dissolved in 10 mL of DI water to form the aqueous phase. M1 (100 mg, 75.7 µmol), BT(Bpin)$_2$ (29.3 mg, 75.7 µmol) , Pd$_2$(dba)$_3$ (1.4 mg, 1.5 µmol), tri(o-tolyl)phosphine (1.2mg, 3.8 µmol), and 78 µL of hexadecane were dissolved in 1 mL of toluene to form the organic phase. Emulsification was achieved by ultrasonication and the reaction was subsequently heated at the respective temperature. At the end of the reaction and after purification via dialysis, a dark blue NP dispersion of PIDTBT (aq) was obtained. Refer to Figure S15 and S16 for representative NMR spectrum of the isolated PIDTBT (aq). **DPPTBT (aq).** The synthesis was performed according to the general procedure described above. NaOH (12mg, 303 µmol) and SDS (75 mg) were dissolved in 10 mL of DI to form the aqueous phase. M2 (103.0 mg, 101 µmol), BT(Bpin)$_2$ ((39.2 mg, 101 µmol), tetrakis(triphenylphosphine)palladium (2.2 mg, 1.0 µmol), and 78 µL of hexadecane were dissolved in 1 mL of toluene to form the organic phase. Emulsification was achieved by ultrasonication and the reaction was subsequently heated at the respective temperature. At the end of the reaction and after purification via dialysis, a dark blue NP dispersion of PDPPTBT(aq) was obtained. Refer to Figure S17 and S18 for representative NMR spectrum for the isolated PIDTBT (aq).

General procedure for conventional polymerization synthesis. Three equivalents of the respective base and a drop of Aliquat 336 were dissolved in 1 mL of DI water in an argon purged Schlenk tube. In a separate tube, the respective monomer M1 or M2 and BT(Bpin)$_2$, the

relevant catalyst and ligand were added at the corresponding stoichiometric amounts for each polymer reaction. The reagents were then dissolved in 4 mL of anhydrous toluene and subsequently bubbled with argon gas for 5 minutes. Next, the organic solution was injected into the Schlenk tube containing the aqueous solution and this was transferred into a heated oil bath at the specified temperature. The reaction was left stirring for 24 hrs under an argon atmosphere. After which the reaction was cooled to room temperature and excess methanol was added into the Schlenk tube to precipitate the polymer. The precipitate was washed with methanol via centrifugation, decanted three times, filtered and dried under vacuum at 40 °C overnight to afford the respective polymer powder. This was dissolved in DCB to obtain solutions of PIDTBT(DCB) and PDPPTBT(DCB). **PIDTBT.** The synthesis was performed according to the general procedure described above. M1 (150 mg, 0.11 mmol), BT(Bpin)$_2$ (44.04 mg, 0.11 mmol), $Pd_2(dba)_3$ (2.08 mg, 2.2 µmol), tris(o-tolyl)phosphine (2.8 mg, 9.1 µmol) and a drop of Aliquat 336 was dissolved in 4 mL of anhydrous toluene, while Na_2CO_3 (36.1 mg, 0.33 mmol) was dissolved in DI water. The reaction proceeded with continuous stirring under an argon atmosphere and was heated at the desired temperature. After methanol precipitation and washing via centrifugation, the polymer was filtered and dried to obtain a dark blue solid powder of PIDTBT. **PDPPTBT.** The synthesis was performed according to the general procedure described above. M2, BT(Bpin)$_2$ (57.12 mg, 0.147 mmol), tetrakis(triphenylphosphine)palladium (3.40 mg, 2.94 µmol) and a drop of Aliquat 336 was dissolved in 4 mL of anhydrous toluene, while K_2CO_3 (62.85 mg, 0.45 mmol) was dissolved in DI water. The reaction proceeded with continuous stirring under an argon atmosphere and was heated at the desired temperature. After methanol precipitation and washing via centrifugation, the polymer was filtered and dried to obtain a dark blue solid powder of PDPPTBT.

Thin-film Characterization. UV-vis absorption spectra of NP dispersions and thin-films were obtained using a Varian Cary UV-Vis-NIR spectrophotometer. The surface morphologies were

measured with a Bruker Multimodal 8 atomic force microscope (AFM) in tapping mode. High-resolution XPS measurements were taken using a microfocused, monochromated Al Kα X – Ray source (1487 eV) and a SPECS Phoibos NAP 150 hemispherical analyser. Scans were taken at normal emission and a pass energy of 30 eV. The thin-film were charge normalised using sp^3 carbon as a reference (the major carbon environment in both the polymers and adventitious carbon) 284.8 eV. Thin-films were spin-coated onto ITO-coated glass substrates, which were attached to sample holders with an adhesive carbon tape, and a top contact was provided to ensure grounding of the ITO coating. The aqueous-processed thin-films were scanned before and after the post-washing step, analysing the same spot on the films in each case.

OFET fabrication and characterization procedure. Top-gate/bottom-contact device architecture was used with Corning glass as a carrier substrate. The glass substrates were cleaned in acetone, 10 wt% DECON 90 solution, DI water and isopropanol under sonication for 15 minutes each, followed by UV-ozone treatment for 5 minutes before device fabrication. Cr/Au bottom electrodes were thermally evaporated under high vacuum (10^{-7} mbar) using a metal shadow mask with a patterned channel width = 1000 µm and length = 60 µm. The substrates were then treated with oxygen plasma for 3 minutes, to generate a hydrophilic surface to ensure sufficient wetting of the aqueous NP dispersions. NP dispersions were deposited on the substrate and left for 60s before spin coating at 500 rpm for 60 s and 6000 rpm for 10s to form the thin-film. The thin-films were annealed at 150°C for 30 minutes and cooled down to room temperature for approximately 30 minutes. The thickness of the resulting polymer semiconductor thin-films were 50-60 nm as measured by contact profilometer (Dektak XT). For the surfactant washing step, Ethanol was dropped over the surface of the thin-film and left for 60s before spin-washing at 6000 rpm for 30s, and the substrates were annealed at 100°C for 5 minutes and left in a vacuum chamber for 15 minutes to allow the removal of any remaining

solvent. For DCB-processed devices, PIDTBT and PDPPTBT were solubilized in anhydrous DCB at 5 mg/mL and stirred overnight at 60 °C, filtered through a 1 µm filter before spin coating at 1000 rpm in a N_2 purged glovebox. The dielectric layer was then spin-coated over the semiconducting thin-films from a solution of poly(methyl methacrylate) (PMMA, M_w = 120,000 Da) in butyl acetate (80 mg/mL), and annealed at 80 °C for 30 minutes. Finally, an aluminum gate electrode (60 nm) was thermally evaporated on the semiconductor/PMMA films using a metal shadow mask. The electrical properties of the OFETs were measured in the dark under ambient condition using an Agilent B1500 semiconductor parameter analyzer. The field-effect mobility (µ) was extracted using equation (1) in the saturation regime from the drain current versus gate voltage sweep:

$$I_{D_{sat}} = C_i \mu_{sat} \frac{W}{2L} (V_{GS} - V_{TH})^2 \qquad (1)$$

where I_D is the drain current in the saturated regime, μ_{sat} is the field-effect mobility, C_i is the capacitance per unit area of the gate dielectric layer, V_G and V_T are gate voltage and threshold voltage and W and L are channel width and length, respectively. The estimated capacitance C_i = 5.78 nF cm^{-2} of PMMA was measured at 100kHz using an Agilent 4284A precision LCR meter. See Figure S9 for a schematic description of the processing procedure of the NP dispersions.

Statistical analysis. The µ values were estimated from transfer curves as representatively shown in Figure S10-S13. The curves were obtained by measurement of a total of 12 transistors fabricated in three independent batches for each processing conditon to obtain individual mean values and the standard deviation. Values for µ are presented in Table S2, and as a box and whisker plot in Figure 3e. The rectangular box is determined by the 25th and 75th percentiles and the whiskers are determined by the 5th and 95th percentiles. The square box is mean and the line is the median.

Representative ^{1}H and ^{13}C NMR in CDCl$_3$ of PIDTBT and PDPPTBT synthesized from mini-emulsion polymerization at reaction temperature of 55 °C and 70 °C respectively.

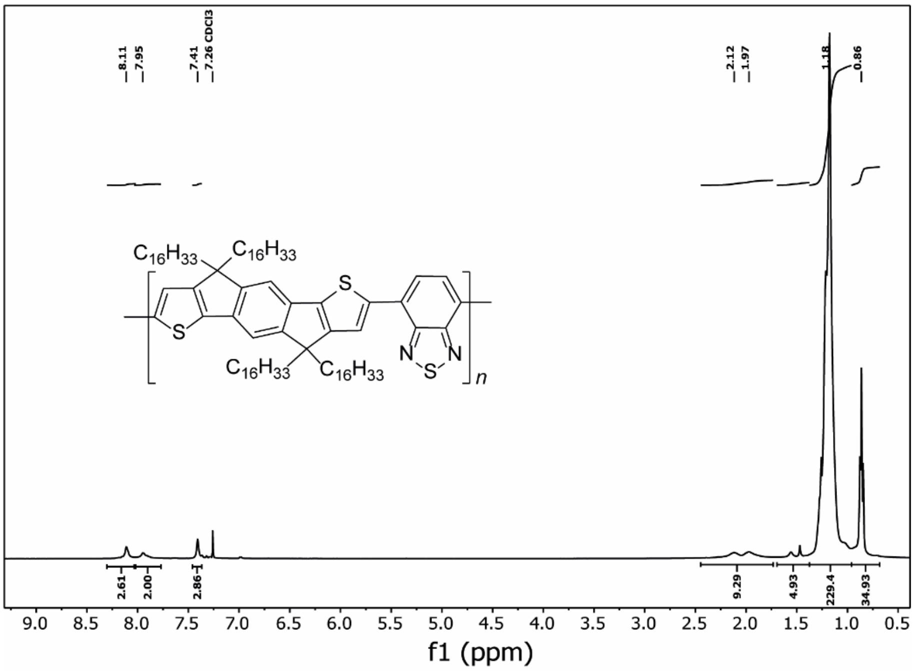

Figure S15. PIDTBT: ^{1}H NMR (400 MHz, CDCl$_3$, 25°C): δ = 8.11 (s, 2H, Ar-H), 7.95 (s, 2H, Ar-H), 7.41 (s, 2H, Ar-H), 1.97 (m, 4H, CH$_2$), 2.12 (m, 4H, CH$_2$) 0.98-1.50 (m, 104 H), 0.67-0.97(m, 20H, CH$_2$ and CH$_3$).

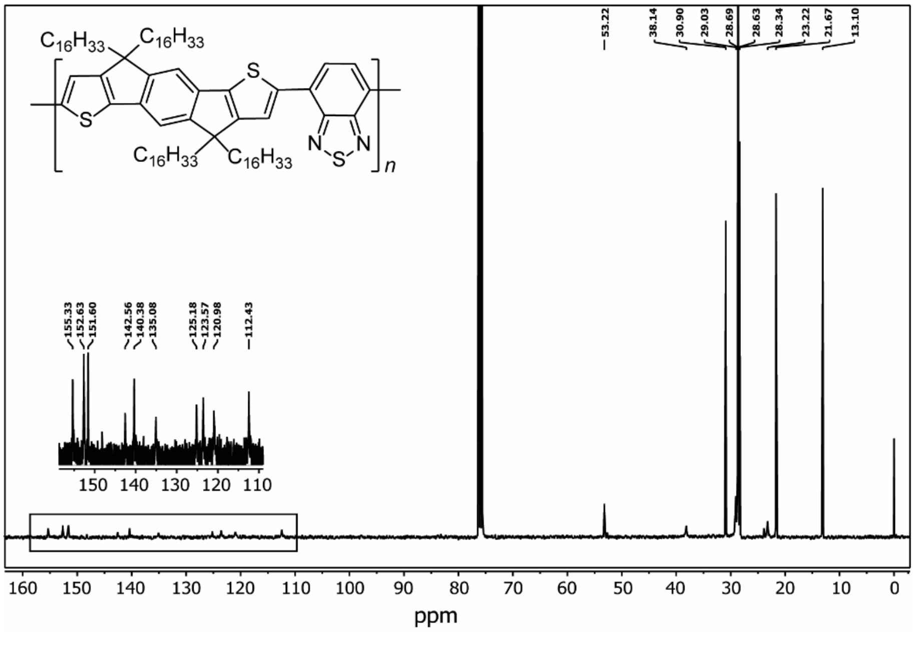

Figure S16. PIDTBT: ^{13}C NMR (400 MHz, CDCl$_3$, 25°C): δ = 155.3, 152. 6, 142.6, 140.3, 135.1, 125.2, 123.6, 121.0, 112.4, 53.2, 38.1, 28.7, 28.6, 28.3, 23.2, 21.6, 13.1.

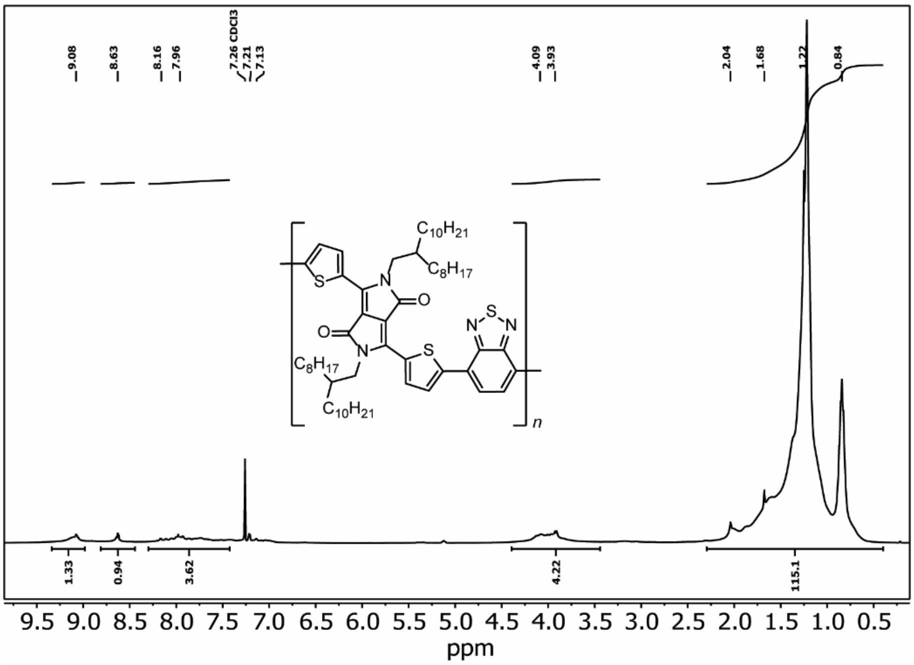

Figure S17. PDPPTBT: ^{1}H NMR (400 MHz, $CDCl_3$, 25°C) δ = 9.08 - 8.63 (m), 8.16 - 7.96 (m), 7.21 - 7.13 (m), 4.09 - 3.93 (m), 2.04 - 0.84 (m).

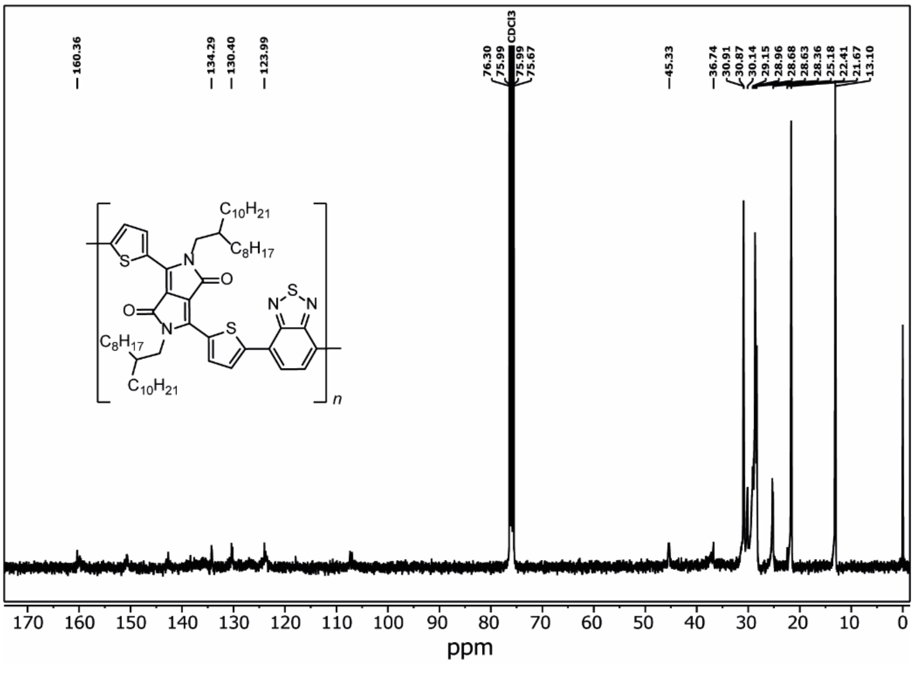

Figure S18. PDPPTBT: ^{13}C NMR (400 MHz, $CDCl_3$, 25°C): δ = 160.36, 134.29, 130.40, 123.99, 76.30, 75.99, 75.67, 45.33, 36.74, 30.91, 30.87, 30.14, 29.15, 28.96, 28.68, 28.63, 28.36, 25.18, 22.41, 21.67, 13.10.
